# Proceedings of the Fourth Annual Conference of the MidSouth Computational Biology and Bioinformatics Society

**DOI:** 10.1186/1471-2105-8-S7-S1

**Published:** 2007-11-01

**Authors:** Dawn Wilkins, Yuriy Gusev, Raja Loganantharaj, Susan Bridges, Stephen Winters-Hilt, Jonathan D Wren

**Affiliations:** 1 Computer & Information Science Department, The University of Mississippi, University, MS, USA; 2 Department of Surgery, Health Sciences Center, The University of Oklahoma, Oklahoma City, Oklahoma 73104, USA; 3 Bioinformatics Research lab, University of Louisiana, Lafayette, LA, USA; 4 Department of Computer Science and Engineering, Mississippi State University, Box 9637, Mississippi State, MS 39762, USA; 5 Department of Computer Science, University of New Orleans, and The Research Institute for Children, 200 Henry Clay Ave., New Orleans, LA 70118, USA; 6 Arthritis and Immunology Research Program, Oklahoma Medical Research Foundation; 825 N.E. 13th Street, Oklahoma City, Oklahoma 73104-5005, USA

## Introduction

The Fourth Annual MidSouth Computational Biology and Bioinformatics Society (MCBIOS-IV) conference was held in New Orleans, Louisiana on February 1^st^–3^rd^, 2007. The conference venue included two locations: the Lindy Boggs Conference facility at the University of New Orleans (UNO); and Dinner/Speaker venues in the French Quarter – Broussard's of New Orleans and The House of Blues. The conference featured three days of technical presentations, with the third day partly devoted to a satellite Conference on Nanopore Cheminformatics with an on-site demo presentation of a nanopore detector (from the Research Institute for Children and UNO Nanopore Cheminformatics/Biophysics Labs). The theme of this year's conference was "Computational Frontiers in Biomedicine".

At MCBIOS 2007, awards for outstanding poster presentations were given to the following students: In Poster Session I, 1^st ^place went to 1st: Mutlu Mete of UALR, 2^nd ^place to William Sanders of MSU and 3^rd ^place to Stephanie Byrum of the UALR. In Poster Session II, 1^st ^place went to Matthew Landry of UNO, 2^nd ^place to Terra Colvin, Jr. of UALR, and 3^rd ^place to Iftekhar Amin of UNO.  1st place for outstanding oral presentation went to Vijayaraj Nagarajan of USM.

## Proceedings summary

This year, 24 out of 31 submitted papers were accepted for inclusion in the proceedings (77%), similar to the number published in last year's Proceedings [1-21]. Each of the papers was peer-reviewed by at least two members of the program committee members and/or external experts in the field. Our goal, as in past years, has been to be inclusive yet rigorous in selecting only high-quality papers. The general themes of this year's proceedings papers fall into several categories:

## Systems biology

One of the most important challenges of current miRNA research is to decipher the "code" of miRNA regulation – to find the connection between miRNA expression and phenotypic changes. Gusev *et al *[22] report the results of a systems biology based analysis of aberrantly expressed miRNAs in five human cancers. Their findings suggest that co-expressed miRNAs collectively provide a systemic compensatory response to the abnormal phenotypic changes in cancer cells by targeting a broad range of functional categories and signaling pathways affected in a particular cancer.

One of the things evident from the Gusev *et al *study is that there is a large body of microarray data that is becoming available for analysis. As such, methods to begin inferring regulatory networks from this data are important. In another paper, Peng Li *et al *compare probabilistic Boolean Network (PBNs) and Dynamic Bayesian Network (DBNs) approaches to correctly inferring regulatory networks [24]. They find that PBNs can reduce the computational complexity, false positive and false negative errors significantly, while DBNs can more accurately derive genetic network interactions, but are more time-consuming.

While microarray technology is steadily improving, it still suffers from noise; hence experiments are repeated several times to reduce error. To reduce the amount of replication necessary, Dozmorov *et al *[23] used F-tests against system-level noise to identify hypervariable genes from time-course microarray experiments. This novel systems-biology approach to biological network reconstruction investigated urothelial cell response to infection with *Enterococcus *bacteria. A complex response was mapped out involving cytoskeletal rearrangement, immune response, modulation of growth and cellular metabolism, and *Wnt *signaling, as well as responses heretofore unrecognized because they involve poorly annotated genes.

## OMICS

Biological analysis spans several different areas from the genome/proteome to the metabolome, collectively referred to as "omics" for the study of different biological bodies. In one study, Schnackenberg *et al *[25] study age-related differences in Sprague-Dawley rats by examining changes in metabolite concentrations in their urine by NMR and UPLC/MS. Their findings are in line with the free-radical theories of aging, as they find a higher concentration of oxidized antioxidants in older rats. They also examine the effects of data normalization procedures and the impact on statistical analyses.

Nagarajan and Elasri [26] use bioinformatics approaches to predict the structure and function of Msa, a novel gene in the human pathogen *S. aureus*. Their combination of methods suggests that Msa is membrane-bound with sites for phosphorylation and protein-binding, suggesting it plays a role in signal transduction, which is consistent with its known activity as a modulator of the protein SarA.

Not all peptide fragments are represented equally in mass spectrometry (MS) experiments. To help predict which peptides might be lost or underrepresented, Sanders *et al. *[27] use artificial neural networks (ANNs) to predict which proteolytic peptides generated by a protein dataset are likely to be detectable by mass spectrometry. The result is an improved method for calculating protein coverage in proteomics experiments and a mechanism for determining if proteins in specific pathways under study are likely to be detected by mass spectrometry.

Bridges et al [28] develop & describe a system, ProtQuant, to provide relative quantification of proteins in high-throughput proteomics samples (MudPIT) using label-free quantification. ProtQuant differs from existing label-free approaches in that it extrapolates the values of missing data points, where possible, from below-threshold identifications. The Java-based tool has a graphical user interface and accepts multiple file formats.

## Cheminformatics

Four papers explore a new transduction-based nanopore detector mechanism. The first [29] introduces the transduction detection method and shows results indicating the applicability to examination of binding in individual molecular complexes in very general circumstances. The next [30] applies this method to the examination of binding for two *DNA-protein binding *interactions: (1) TBP – TATA receptor binding, and (2) HIV Integrase – HIV DNA Terminus binding. The method is also effective at detecting *DNA-DNA binding *interactions that occur with annealing of DNA single-strand overhangs [31] as well as *protein-protein binding *interactions for the medically important case of antibody-antigen interactions [32].

## Machine learning based pattern recognition

Pattern recognition is a critical part of making sense of the high-throughput data gathered in modern biomedical experiments. Four papers explore the development of machine learning based pattern recognition methods and their application to resolving complex nanopore-transduction detector signals. The first [33] describes a new Support Vector Machine (SVM) based method for *clustering *(unsupervised learning) – a marked departure from the standard supervised-learning approach to SVMs. The author's objective was to have a powerful, non-parametric, method for phase tracking on nanopore transduction signals, a key requirement for extracting binding kinetics from channel current signals. They also describe a new form of Hidden Markov Model (HMM) that has the strengths of the much more complex HMM-with-Duration (HMMwD) models, but at a computational cost approximating the simpler HMM [34]. The goal is to apply this method in a real-time pattern recognition informed sampling process on the nanopore detector. The third paper, [35], examines learning on exact HMMwD models and their use in two-state signal decomposition. The fourth paper, [36], explores (i) non-standard HMM implementations for improved feature extraction and SVM classification performance, (ii) SVM classification improvements resulting from introduction of a single "spike density" feature; and (iii) SVM improvements resulting from introducing a huge family of HMM transition probability features subsequently pruned by an AdaBoost Selection process.

## Microarray studies

Rather than focus on refinement of methods, several authors report the effectiveness and utility of existing bioinformatics approaches to better understand a biological system. For example, Nan Mei *et al *[37] studied gene expression changes in the livers of riddelline-treated Big Blue rats. Standard analysis methods and popular pathway analysis software was used to determine that the genes differentially expressed with significance were mainly involved in cancer, tissue development, apoptosis, cellular growth and proliferation, and others. The study helped elucidate the mechanisms involved in toxicity and carcinogenesis due to exposure to riddelline.

Guo *et al*. [38] used microarrays in conjunction with pathway analysis software to test the hypothesis that Pyrrolizidine alkaloids (PAs), common in many plants, cause liver toxicity and/or cancer in experimental animals. They found that genes within carcinogenic pathways were disproportionately altered, supporting their hypothesis.

Circadian rhythms are generally associated with the sleep/wake cycle, but also regulate many activities and affect the expression pattern of practically all genes. Time-course microarrays can potentially detect this baseline oscillation, which Ptitsyn and Gimble [39] use in an interesting study on the leptin signaling system. This system is a major regulator of energy metabolism, responsible to the sensation of satiety after a meal. They observe tissue-specific alternative polyadenylation of SOCS3 transcripts, whereby alternative transcripts different by the length of a 3' UTR oscillate in counter-phase. This study suggests a mechanism that can provide a constant abundance of transcript and volume of cytokine signal transduction regardless of circadian time.

## Genomic analysis

Identification of DNA binding sites for transcription factors (motifs) is important for a complete understanding of co-regulation of gene expression, but has proven to be quite challenging. Das and Dai [40] review previously published algorithms for DNA motif finding. The algorithms reviewed are string-based, probabilistic, and machine learning techniques that fall into three major classes: Those that use promoter sequences of co-regulated genes from single genome, phylogenetic footprinting, and a hybrid of the two. Although there has been substantial progress in this area within recent years and algorithms work reasonably well for prokaryotic organisms, success with motif finding in eukaryotes has been more elusive.

Loganantharaj *et al *[41] have proposed a general methodology for validating the effectiveness of phylogenetic profiling, using the Gene Ontology as the gold standard for validating functional similarity among the genes in each cluster. They demonstrated that phylogenetic profiling technique showed poor performance in functional prediction in human and mouse. However, their empirical study shows strong support for few cohesive functional groups in each phylogenetic cluster. They concluded that phylogenetic profiling is still a very useful technique for predicting function of an unknown protein sequence.

Pirooznia *et al *[42] report the results of a large-scale EST sequencing project for the earthworm, *Eisenia fetida*, which is often used in toxicology studies. They describe the sequencing and analysis of 3,144 new ESTs.

## Miscellaneous

The insertion of new or altered genes into genomes is a key step in many functional analysis studies, and it is important to determine how many copies of each of these transgenes are present. Yuan *et al *[43] report the development of a new statistical approach that facilitates a more accurate transgene copy number estimation.

Ding *et al *[44] propose a new algorithm for divisive clustering, which is similar to bisecting *k*-median, but which uses statistical spatial depth to identify the "center" of a cluster. A new subcluster selection rule, Relative Average Depth, is also introduced. In data sets that are noisy or have high dimension and low sample size, which is common in gene expression data sets, the bisecting *k*-spatial median algorithm does well compared to the component-wise bisecting *k*-median algorithm.

Cancer diagnosis usually begins with histopathological examinations of tissue biopsies. These evaluations are usually somewhat subjective and the growing number of such images has provides an opportunity to test automated approaches to tissue sample categorization. Mete *et al *[45] report a method for automated analysis of squamous cell carcinomas using a SVM. They report a classification accuracy of 96% on their test set, which is quite promising for the future of histopathology.

## Future meetings

The fifth annual MCBIOS Conference will be held in Oklahoma City, Oklahoma in the Cox Convention Center in downtown Oklahoma City on February 23^rd ^and 24^th^, 2008. Our web site, , contains further information on the society and future meetings. MCBIOS is a regional affiliate of the International Society for Computational Biology .

## Competing interests

The authors declare that they have no competing interests.

## Authors' contributions

All authors served as co-editors for these proceedings, with JDW serving as Senior Editor. All authors helped write this editorial.
